# The triglyceride glucose-body mass index and the risk of gestational diabetes mellitus: a systematic review and meta-analysis

**DOI:** 10.3389/fnut.2026.1833116

**Published:** 2026-06-09

**Authors:** Jian Sun, Yanxu Chen, Jinying Chen, Tongling Zhao, Hezeng Dong, Hui Wang, Zheng Nan, Bo Dai

**Affiliations:** 1 Changchun University of Chinese Medicine, Changchun, China; 2 College of Science and Technology Changchun, Changchun, China; 3 The Affiliated Hospital of Changchun University of Chinese Medicine, Changchun, China

**Keywords:** gestational diabetes mellitus, meta-analysis, risk factor, system review, triglyceride glucose-body mass index

## Abstract

**Objective:**

This study aimed to systematically evaluate the association of triglyceride-glucose body mass index (TyG-BMI) with the risk of gestational diabetes mellitus (GDM).

**Methods:**

A systematic search was conducted in PubMed, Embase, Web of Science, the Cochrane Library, China National Knowledge Infrastructure, Wanfang Data, and Chinese Science and Technology Journal Database from inception to 31 March 2026 for cohort studies investigating the association between TyG-BMI and GDM risk. Two reviewers independently conducted literature screening, data extraction and study quality assessment using the Newcastle-Ottawa Scale (NOS). Meta-analysis was performed with Stata 16.0 for heterogeneity test, sensitivity analysis and publication bias assessment, and Review Manager 5.4.1 for effect size pooling and subgroup analyses. Heterogeneity across studies was assessed using the Cochrane Q-test and *I*^2^ statistic. A fixed-effects model was used for effect size pooling if heterogeneity was low (*P* > 0.1, *I*^2^ ≤ 40%); a random-effects model was adopted for significant heterogeneity (*P* < 0.1, *I*^2^ ≥ 50%). Subgroup analyses were stratified by study design, maternal age and sample size to explore heterogeneity sources. Sensitivity analyses were performed to examine result stability, and publication bias was assessed by funnel plot and Egger's regression test.

**Results:**

This meta-analysis included a total of 6 cohort studies with 52,877 participants. The overall pooled OR was 2.13 (95% CI: 1.26–3.61, *P* = 0.005, *I*^2^ = 97%). Subgroup analyses showed significant positive associations in prospective cohorts (OR = 2.55, 95% CI: 1.82–3.58, *P* < 0.0001, *I*^2^ = 49%), women aged ≤ 32 years (OR = 2.12, 95% CI: 1.53–2.95, *P* < 0.0001, *I*^2^ = 45%), and large-sample studies (≥1,000 participants, OR = 2.39, 95% CI: 1.77–3.22, *P* < 0.0001, *I*^2^ = 70%). A marginally significant association was found in retrospective cohorts (OR = 1.78, 95% CI: 1.00–3.17, *P* = 0.05, *I*^2^ = 91%), whereas no significant associations were identified in women aged >32 years (OR = 2.08, 95% CI: 0.89–4.83, *P* = 0.09, *I*^2^ = 99%) or small-sample studies (<1,000 participants, OR = 1.68, 95% CI: 0.54–5.22, *P* = 0.37, *I*^2^ = 69%). No significant between-subgroup differences were observed across all analyses. Sensitivity analyses indicated stable results, with *I*^2^ reduced from 97% to 60% following the exclusion of one study. Funnel plot and statistical tests showed no significant publication bias; however, these assessments have limited reliability when fewer than 10 studies are included and should be interpreted with caution.

**Conclusions:**

This exploratory meta-analysis suggests that increased early-pregnancy TyG-BMI was significantly associated with a higher risk of GDM. TyG-BMI may serve as a potential early predictive biomarker for GDM, although further large-scale prospective studies are warranted to validate these results and identify potential effect modifiers.

**Systematic review registration:**

https://www.crd.york.ac.uk/PROSPERO/view/CRD420261291099, identifier: CRD420261291099.

## 1 Introduction

Gestational diabetes mellitus (GDM), defined as glucose intolerance first diagnosed during pregnancy, has evolved into a major public health concern worldwide. The prevalence of GDM is rising, ranging from 12.8% to 16.7% among Chinese pregnant women and approximately 14% worldwide (1, 2). This metabolic disorder not only increases the risk of immediate adverse pregnancy outcomes including hypertensive disorders of pregnancy, macrosomia, and fetal distress, but also confers long-term metabolic risks to mothers and offspring (3). Specifically, mothers are at an increased risk of type 2 diabetes and cardiovascular disease, whereas offspring have a higher likelihood of obesity and impaired glucose tolerance (IGT) in childhood and adolescence (4, 5). Timely identification of high-risk populations and early intervention are crucial for reducing these short- and long-term complications. At present, the gold standard for GDM diagnosis remains the 75-g oral glucose tolerance test (OGTT), which is routinely administered at 24–28 weeks of gestation (6). However, this diagnostic window falls in mid-to-late pregnancy, leaving limited time for effective preventive interventions to modify the disease course (7). Thus, there is an urgent need for simple, accessible, and reliable early predictive markers to identify GDM risk as early as the first trimester of pregnancy.

Insulin resistance (IR) is a core pathophysiological mechanism underlying GDM development (8). Although the hyperinsulinemic-euglycemic clamp represents the gold standard for measuring IR, its complicated procedure, high expense, and invasive character restrict its widespread use in routine prenatal screening (9). As a result, surrogate metabolic indices integrating easily measurable parameters have garnered growing attention. The triglyceride-glucose (TyG) index, derived from fasting triglyceride (TG) and fasting plasma glucose (FPG) levels, has been confirmed as a reliable indicator of IR and shows favorable predictive potential for GDM (10, 11). Building on this, the triglyceride glucose-body mass index (TyG-BMI) incorporates pre-pregnancy BMI into the TyG index, integrating the effects of glucose metabolism, dyslipidemia and obesity, all of which are key contributors to GDM risk (12, 13). Recent cohort studies have consistently shown that higher early-pregnancy TyG-BMI is associated with an increased risk of GDM (14–16). However, these studies display substantial heterogeneity in design, population, sample size, and confounding adjustment. No systematic review or meta-analysis has yet synthesized the evidence to verify the overall association and clarify key sources of heterogeneity.

Given this research gap, this study aimed to evaluate the association between early-pregnancy TyG-BMI and GDM risk, explore potential sources of between-study heterogeneity, and assess the clinical value of TyG-BMI as an early predictive marker for GDM. Our findings intend to provide evidence-based support for early risk stratification and preventive strategies in clinical practice.

## 2 Methods

This study was registered on PROSPERO (CRD420261291099) and conducted in line with the PRISMA 2020 guidelines (17). The PRISMA checklist is provided in Supplementary Material S1.

### 2.1 Database and search strategy

This study performed systematic literature searches in PubMed, Embase, Web of Science, the Cochrane Library, China National Knowledge Infrastructure, Wanfang Data, and Chinese Science and Technology Journal Database up to 31 March 2026. Literature searches were independently performed by two investigators (SJ, CYX). Key search terms included “diabetes, gestational,” “gestational diabetes mellitus,” “GDM,” “triglyceride-glucose body mass index,” “TyG-BMI,” and “cohort study.” The detailed search strategy is provided in Supplementary Material S2.

### 2.2 Study selection criteria

Study selection criteria were established according to the PECOS framework.

#### 2.2.1 Participants

Pregnant women in early pregnancy at 6–14 weeks of gestation with no previous diagnosis of type 1 diabetes, type 2 diabetes, or gestational diabetes in a prior pregnancy.

#### 2.2.2 Exposure

Studies reporting the TyG-BMI index were included. The formula for calculation is as follows:

(1) TyG = ln [triglycerides (mg/dL) × glucose (mg/dL)/2];
(2) BMI = body mass (kg)/height^2^ (m^2^);
(3) TyG-BMI = TyG × BMI.

Note: ln = natural logarithm.

#### 2.2.3 Comparators

Studies comparing participants in the highest category (e.g., top quartile/tertile) versus the lowest category (e.g., bottom quartile/tertile) of TyG-BMI levels, with the lowest category serving as the reference group, were included.

#### 2.2.4 Outcomes

The primary outcome was newly diagnosed GDM with clear diagnostic criteria reported. Included studies must provide the odds ratio (OR) and 95% confidence interval (CI) for the association between TyG-BMI and newly diagnosed GDM.

#### 2.2.5 Study design

Eligible studies were prospective or retrospective cohort studies published in English or Chinese in peer-reviewed journals with full-text availability.

### 2.3 Exclusion criteria

#### 2.3.1 Types of participants

Participants were excluded if they had pregestational diabetes mellitus, multiple pregnancies, chronic liver or kidney disease, endocrine disorders including thyroid dysfunction, or treatment with medications known to affect glucose or lipid metabolism.

#### 2.3.2 Exposure

Studies using other metabolic indices or measuring TyG-BMI in the second or third trimester were excluded.

#### 2.3.3 Comparators

Studies without a defined reference group or using arbitrary cut-offs not validated for GDM prediction were excluded.

#### 2.3.4 Outcome measures

Studies reporting only other pregnancy outcomes without GDM data or using non-standard GDM diagnostic criteria were excluded.

#### 2.3.5 Type of study type

Case-control, cross-sectional, case-report, review, meta-analysis, letter, editorial, and conference abstract studies were excluded, as well as those with insufficient data to derive OR and 95% CI.

### 2.4 Literature screening and data extraction

Two researchers (SJ, CYX) constructed the literature database using EndNote X9 software. Literature screening and data extraction were conducted independently and in duplicate by two reviewers to minimize bias. They employed cross-validation to confirm the inclusion and exclusion criteria for studies. Disagreements were arbitrated by a third researcher (DB), with final consensus reached through discussion. Two additional researchers (ZTL, DHZ) independently extracted key information from the included studies, including Study, Country, Study design, Gestational trimester, Age, Number of participants, TyG-BMI, OR (95% CI), Outcome type, and Variables adjusted. Data extraction was validated through cross-checking.

### 2.5 Quality assessment

All included cohort studies were quality-assessed using the Newcastle-Ottawa Scale (NOS) (18). This scale evaluates three domains: subject selection, group comparability, and outcome assessment. The total score ranges from 1 to 9 points, and studies with a score above 6 were regarded as high-quality. Two independent researchers (SJ, DB) performed the assessment, and any discrepancies were resolved by consensus.

### 2.6 Statistical analysis

Statistical analyses of GDM outcomes were performed using Review Manager 5.4.1 and Stata 16 software. The OR and 95% CI were used as effect measures to quantify the association between TyG-BMI and GDM risk. For categorical TyG-BMI, pooled ORs were calculated by comparing the highest versus lowest categories or quartiles.

Heterogeneity across studies was evaluated by the Cochrane Q-test with a significance level of *P* < 0.1. The *I*^2^ statistic with 95% CI was used to quantify the magnitude of heterogeneity. The interpretation of *I*^2^ was based on the Cochrane Handbook for Systematic Reviews of Interventions (7th edition): values of 0%−40% represented low or no heterogeneity, 30%−60% represented moderate heterogeneity, 50%−90% represented substantial heterogeneity, and 75%−100% represented considerable heterogeneity (19). Subgroup analyses stratified by study design, maternal age, and sample size were performed to identify potential sources of heterogeneity. Pooled ORs were calculated for each subgroup, and between-subgroup differences were examined using the Q-test with *P* < 0.05 defined as statistically significant. A fixed-effects model was adopted when heterogeneity was low (*P* > 0.1 and *I*^2^ ≤ 40%). If significant heterogeneity was present (*P* < 0.1 or *I*^2^ ≥ 50%), predefined subgroup analyses were first conducted to explore sources of variation, and a random-effects model was then used for the pooled analysis.

Sensitivity analysis was conducted to test the robustness of the main findings by sequentially excluding individual studies and recalculating the pooled OR. Funnel plots and Egger's test were used to assess publication bias, with *P* < 0.05 indicating statistical significance.

## 3 Results

### 3.1 Literature screening results

**Figure 1:**
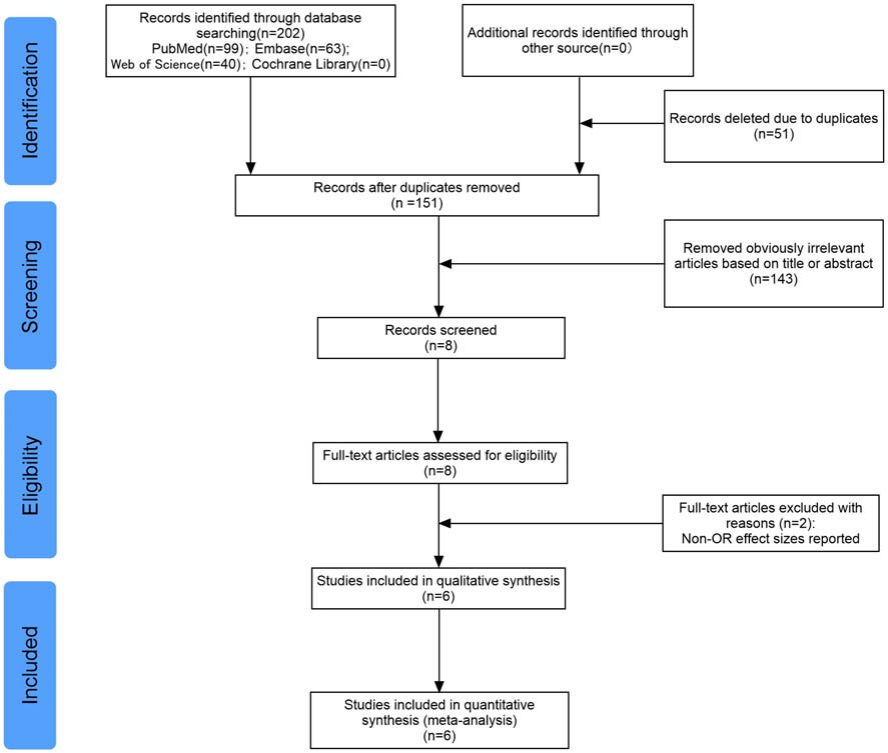
Flowchart of study selection.

A total of 202 records were initially identified through database searching, including 99 from PubMed, 63 from Embase, and 40 from Web of Science. Following the removal of 51 duplicates, 151 records remained. Of these, 143 obviously irrelevant articles were excluded through title and abstract screening. The remaining 8 articles were then assessed in full text, from which 2 studies were excluded due to the absence of odds ratio effect sizes. Finally, 6 studies were included in this meta-analysis (13, 16, 20–23). The literature screening process is depicted in Figure 1.

### 3.2 Basic information of the included studies

All cohort studies included in this meta-analysis were published in 2025, with sample sizes ranging from 600 to 46,992 participants. A total of 52,877 individuals were enrolled across the included studies, among whom 7,060 were newly diagnosed with GDM. Currently, no universally accepted optimal threshold for TyG-BMI to predict GDM has been established in the literature, as cutoff values vary by study population, gestational age, and ethnicity. Accordingly, all included studies adopted quantile-based stratification according to their respective populations. Participants in all studies were early pregnant women at 6–14 weeks of gestation. The mean or median baseline metabolic parameters across study populations were within the following ranges: pre-pregnancy BMI 18.94–25.73 kg/m^2^, fasting triglyceride levels 0.83–1.40 mmol/L, and fasting plasma glucose levels 4.50–4.83 mmol/L. Detailed characteristics of each included study are summarized in Table 1.

**Table 1:** Basic characteristics of the included studies.

Number	Reference	Country	Study design	Gestational trimester	Age (year)	Number of participants	TyG–BMI index condition	OR (95%CI)	Type of outcome	Variables adjusted
1	Junxiang Gao et al. (20)	China	Prospective cohort study	First trimester (<12 weeks)	Median age: 30 years	1,450	Categorized	1.888 (1.248–2.871)	Gestational Diabetes Mellitus	Age, prepregnant weight gain rate, diastolic blood pressure, fasting insulin, Low–density lipoprotein cholesterol and Family history of diabetes mellitus.
2	Lirui Zhang et al. (22)	China	Prospective cohort study	First trimester (6–13+6 weeks)	Average age: 34.52 ± 3.97 years	46,992	Categorized	2.91 (2.53–3.36)	Gestational Diabetes Mellitus	Age, multiparity, pre–pregnancy BMI, family history of diabetes, history of GDM, education level, first–trimester Total Cholesterol and Low–Density Lipoprotein Cholesterol levelsight and obesity.
3	Xiaomin Liang et al. (16)	South Korean	Prospective cohort study	First trimester (10–14 weeks)	Average age: 32.07 ± 3.80 years	588	Categorized	3.73 (0.99–14.12)	Gestational Diabetes Mellitus	Age, parity, Aspartate Aminotransferase, insulin, Low–Density Lipoprotein Cholesterol, hepatic steatosis, Gamma–Glutamyl Transferase, Alanine Aminotransferase, and High–Density Lipoprotein Cholesterol
4	Yezi Hu et al. (21)	China	Retrospective cohort study	First trimester (9–13 weeks)	Average age: 30.1 ± 3.5years	2,111	Categorized	1.8 (1.28–2.52)	Gestational Diabetes Mellitus	Age, Aspartate Aminotransferase, Alanine Aminotransferase, Total Cholesterol, Total Bilirubin, Direct Bilirubin, Creatinine and High–Density Lipoprotein Cholesterol.
5	Zhaoran Meng et al. (13)	China	Retrospective cohort study	First trimester (10–13+6 weeks)	Median age: 32 years	1,136	Categorized	3.27 (1.92–5.59)	Gestational Diabetes Mellitus	Age, primipara, artificial conception, and family history of hypertension and diabetes mellitus.
6	Pinghua Li et al. (23)	China	Retrospective cohort study	First trimester (10–14 weeks)	Median age: 35 years	600	Categorized	1.109 (1.029–1.038)	Gestational Diabetes Mellitus	Age, pre–pregnancy BMI, Fasting Plasma Glucose, Total Cholesterol and Low–Density Lipoprotein Cholesterol.

### 3.3 Quality assessment

Quality assessment of the included studies was performed using the NOS for cohort studies, which evaluates methodological quality across three core domains: cohort selection, cohort comparability, and outcome assessment. A binary scoring system was applied, with 1 assigned to items meeting quality requirements and 0 to those that did not, yielding a maximum total score of 9 points. Among the 6 included cohort studies, 2 scored 9 points, 3 scored 8 points, and 1 scored 7 points, indicating overall high methodological quality. All included studies met the design specifications for cohort comparability and most items for cohort selection. Minor quality limitations were observed mainly in the representativeness of the exposed cohort and adequacy of follow-up. Detailed NOS assessment results are presented in Table 2.

**Table 2:** Details of quality evaluation via the Newcastle–Ottawa Scale.

Reference	Selection of cohorts			Comparability of cohorts			Outcome of cohorts			Total
	Representativeness of the exposed cohort	Selection of the non–exposed cohort	Ascertainment of exposure	Outcome not present at baseline	Control for age	Control for other confounding factors	Assessment of outcome	Sufficient follow–up duration	Adequacy of follow–up of cohorts	
Junxiang Gao et al. (20)	1	1	1	1	1	1	1	1	1	9
Lirui Zhang et al. (22)	1	1	1	1	1	1	1	1	1	9
Xiaomin Liang et al. (16)	0	1	1	1	1	1	1	1	1	8
Yezi Hu et al. (21)	0	1	1	1	1	1	1	1	0	7
Zhaoran Meng et al. (13)	0	1	1	1	1	1	1	1	1	8
Pinghua Li et al. (23)	0	1	1	1	1	1	1	1	1	8

### 3.4 Meta-analysis results

**Figure 2:**
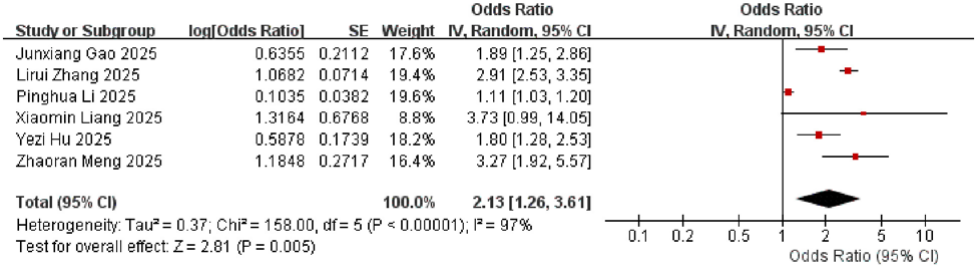
Forest plot of the association between early-pregnancy TyG-BMI and GDM risk.

A total of 6 cohort studies encompassing 52,877 participants were incorporated into this meta-analysis. The overall pooled OR was 2.13 (95% CI: 1.26–3.61, *P* = 0.005; *I*^2^ = 97%), which suggested a significant positive association between elevated early-pregnancy TyG-BMI levels and a higher risk of GDM. All included studies exhibited a consistent direction of effect, alongside substantial between-study heterogeneity (*I*^2^ = 97%, *P* < 0.0001). A random-effects model was adopted to mitigate this high degree of heterogeneity. The forest plot is presented in Figure 2.

**Figure 3:**
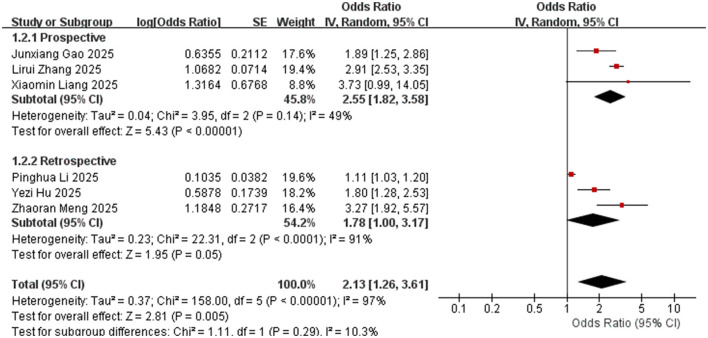
Subgroup analysis by study design.

**Figure 4:**
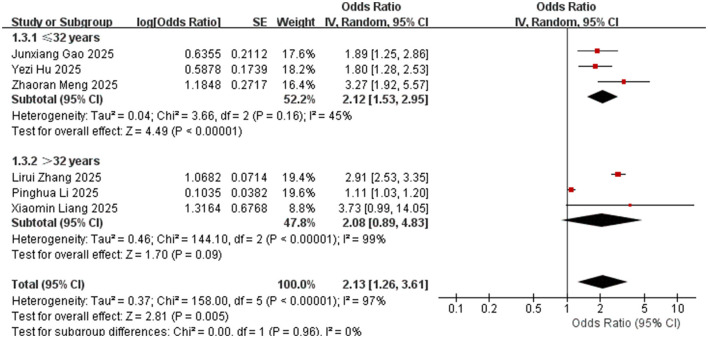
Subgroup analysis by maternal age.

**Figure 5:**
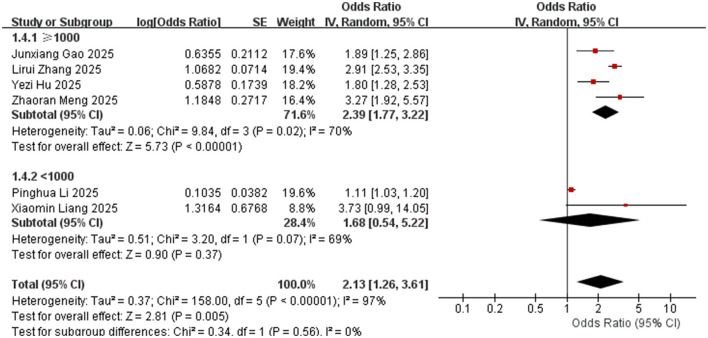
Subgroup analysis by sample size.

To explore potential contributors to heterogeneity, subgroup analyses were carried out according to study design, maternal age, and sample size. For stratification by study design, the prospective cohort subgroup showed a pooled OR of 2.55 (95% CI: 1.82–3.58, *P* < 0.0001) with moderate heterogeneity (*I*^2^ = 49%). The retrospective cohort subgroup yielded a pooled OR of 1.78 (95% CI: 1.00–3.17, *P* = 0.05) with high heterogeneity (*I*^2^ = 91%). No significant subgroup difference was observed (χ^2^ = 1.11, df = 1, *P* = 0.29; *I*^2^ = 10.3%). Corresponding forest plots are presented in Figure 3. For stratification by maternal age, the younger subgroup (≤ 32 years) showed a pooled OR of 2.12 (95% CI: 1.53–2.95, *P* < 0.0001) with moderate heterogeneity (*I*^2^ = 45%). The older subgroup (>32 years) yielded a pooled OR of 2.08 (95% CI: 0.89–4.83, *P* = 0.09) with extremely high heterogeneity (*I*^2^ = 99%). No significant between-subgroup difference was detected (χ^2^ = 0.00, df = 1, *P* = 0.96; *I*^2^ = 0%). Corresponding forest plots are presented in Figure 4. For stratification by sample size, the large-sample subgroup (≥1,000 participants) showed a pooled OR of 2.39 (95% CI: 1.77–3.22, *P* < 0.0001) with moderate heterogeneity (*I*^2^ = 70%). The small-sample subgroup (<1,000 participants) yielded a pooled OR of 1.68 (95% CI: 0.54–5.22, *P* = 0.37) with high heterogeneity (*I*^2^ = 69%). No significant subgroup difference was found (χ^2^ = 0.34, df = 1, *P* = 0.56; *I*^2^ = 0%). Corresponding forest plots are presented in Figure 5. Across all 6 included studies, the overall pooled OR remained 2.13 (95% CI: 1.26–3.61, *P* = 0.005) with persistent high heterogeneity (*I*^2^ = 97%).

A dose-response trend was observed, with GDM risk increasing progressively as TyG-BMI levels increased. However, a unified clinically actionable cut-off value could not be established due to the limited number of studies and lack of individual participant data.

### 3.5 Publication bias analysis

**Figure 6:**
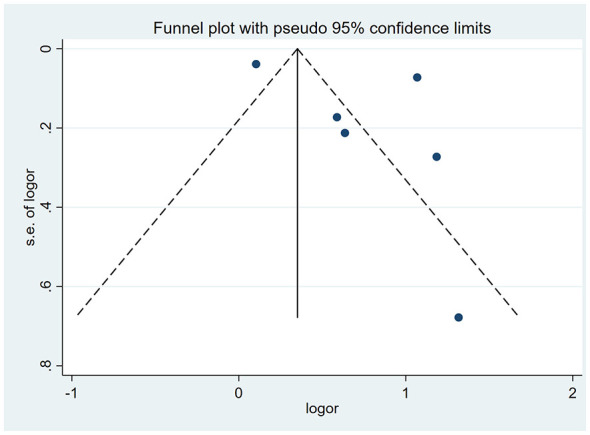
Funnel plot for publication bias.

Visual inspection of the funnel plot showed a generally symmetrical distribution of the 6 included studies. Begg's test (*z* = 0.56, *P* = 0.573) and Egger's test (*t* = 1.23, *P* = 0.285) were both non-significant. However, both funnel plot evaluation and statistical tests for publication bias have limited reliability when fewer than 10 studies are included (24). Therefore, we cannot definitively rule out the potential risk of publication bias. The funnel plot is presented in Figure 6.

### 3.6 Sensitivity analysis

A leave-one-out sensitivity analysis was conducted to evaluate the stability of the pooled effect size. Exclusion of any individual study resulted in pooled ORs ranging from 1.91 to 2.43, all of which remained statistically significant (*P* < 0.0001), exception of the exclusion of Li et al. (23), where *P* = 0.04. Notably, removal of the study by Li et al. (23) yielded a pooled OR of 2.43 (95% CI: 1.84–3.21) and a substantial reduction in heterogeneity (*I*^2^ = 60%), confirming that this study was the primary contributor to the initial high heterogeneity (*I*^2^ = 97%). Exclusion of each of the other individual studies did not markedly alter the overall effect size or heterogeneity, with pooled ORs ranging from 1.91 to 2.22 (all *P* < 0.0001) and *I*^2^ values ranging from 87% to 97%. These results support the reliability and stability of the overall association between elevated TyG-BMI and increased GDM risk, as the direction and statistical significance of the findings were consistent across all sensitivity analyses. Detailed results of the sensitivity analysis are shown in Table 3.

**Table 3:** Summary of sensitivity analysis.

Dataset excluded	OR	95%CI	I^2^%	*P* for effect
Junxiang Gao 2025	2.19	[1.20, 4.00]	97	*P* < 0.001
Lirui Zhang 2025	1.91	[1.21, 3.00]	87	*P* < 0.001
Xiaomin Liang 2025	2.02	[1.16, 3.51]	97	*P* < 0.001
Yezi Hu 2025	2.22	[1.20, 4.11]	97	*P* < 0.001
Zhaoran Meng 2025	1.96	[1.10, 3.48]	97	*P* < 0.001
Pinghua Li 2025	2.43	[1.84, 3.21]	60	*P* = 0.04

## 4 Discussion

GDM is a major global public health issue characterized by increasing prevalence and earlier onset (25). As a metabolic condition triggered by excessive pregnancy-related IR and inadequate pancreatic β-cell compensation (26), GDM is associated with acute adverse pregnancy outcomes such as macrosomia, preterm birth, and preeclampsia, as well as long-term metabolic sequelae in mothers and their offspring (4, 27). During normal pregnancy, adaptive IR supports fetal nutrient supply, but its dysregulation combined with pre-pregnancy obesity, advanced maternal age, or genetic predisposition can disrupt glucose homeostasis and lead to GDM (28). Because routine screening is commonly performed at 24–28 weeks of gestation, early and reliable predictive markers are urgently needed for timely risk stratification and intervention.

The TyG index is a well-validated non-invasive surrogate for IR that avoids the complexity and high cost of the hyperinsulinemic-euglycemic clamp (29). BMI, a classic indicator of adiposity and metabolic burden, is also an independent risk factor for GDM (30). The TyG-BMI index combines the IR reflection of the TyG index and the metabolic load represented by BMI, providing a more comprehensive marker of gestational metabolic dysfunction (20). Previous investigations have indicated that TyG-BMI outperforms TyG or BMI alone in predicting metabolic diseases, and its dependence on standard prenatal assessments and pre-pregnancy BMI makes it well-suited for early gestational screening (31).

This meta-analysis confirmed a significant positive association between early-pregnancy TyG-BMI and GDM risk, which is biologically reasonable. Elevated TyG-BMI reflects concurrent glucose and lipid disorders and excess adiposity, both of which are key pathogenic factors of GDM. High triglycerides induce lipotoxicity and impair insulin signaling, while excess visceral fat related to high BMI releases pro-inflammatory cytokines that further reduce insulin sensitivity (32, 33). Chronic low-grade inflammation is now recognized as a central driver of GDM pathogenesis, with dysregulated pro-inflammatory signaling exacerbating IR and contributing to disease progression. Recent evidence has further linked this inflammatory state to altered expression of long non-coding RNAs (lncRNAs), including metastasis-associated lung adenocarcinoma transcript 1 (MALAT1), maternally expressed gene 3 (MEG3), and X inactive-specific transcript (XIST). These lncRNAs are significantly downregulated in GDM, and their reduced expression is closely associated with elevated proinflammatory cytokines, aggravated insulin resistance, and impaired pancreatic β-cell function, thereby contributing to the pathogenesis and progression of GDM. These lncRNAs are inversely correlated with both IR and proinflammatory cytokines including TNF-α, IL-6, and IL-1β (34). Moreover, sustained metabolic stress reflected by elevated TyG-BMI can also induce glucotoxicity and lipotoxicity, leading to pancreatic β-cell dysfunction and insufficient insulin secretion, which further promotes the progression from IR to GDM. These metabolic and inflammatory abnormalities are aggravated by early pregnancy hormonal changes, increasing susceptibility to GDM (35). By integrating both modifiable and non-modifiable risk factors, TyG-BMI serves as a reliable and practical early predictive marker for GDM in clinical practice.

This meta-analysis of 6 cohort studies including 52,877 participants confirmed a significant positive association between elevated early-pregnancy TyG-BMI and GDM risk, with a pooled OR of 2.13 (95% CI 1.26–3.61, *P* = 0.005). This finding supports the biological mechanisms described above and is consistent with results from individual studies. For instance, Xiaomin Liang et al. (16) reported an OR of 3.73 in a Korean cohort, and Lirui Zhang et al. (22) observed an OR of 2.91 in a large Chinese population, both supporting the predictive ability of TyG-BMI. The high heterogeneity (*I*^2^=97%) may be attributed to clinical and methodological differences rather than statistical grouping alone, including variations in GDM diagnostic criteria, gestational weeks at TyG-BMI measurement, confounder adjustment sets, population characteristics, and TyG-BMI grouping strategies across studies. These clinical factors may serve as the primary sources of heterogeneity. Subgroup analyses further showed that the association was more prominent in prospective cohorts (OR = 2.55, *I*^2^ = 49%), younger women (≤ 32 years, OR = 2.12, *I*^2^ = 45%), and large-sample studies (OR = 2.39, *I*^2^ = 70%). The stronger effect in prospective studies may reflect more standardized exposure assessment and lower recall bias. The significant association among younger women suggests that TyG-BMI can detect early metabolic disorders even in groups usually regarded as low-risk. By contrast, the non-significant results in older women (>32 years, OR = 2.08, *P* = 0.09) and small-sample studies (OR = 1.68, *P* = 0.37) may be due to extremely high heterogeneity (*I*^2^ = 99%) and insufficient statistical power, respectively. Importantly, no significant between-group differences were found, indicating that the association between TyG-BMI and GDM remains consistent across different populations and study designs.

Sensitivity analysis supported the stability and consistency of our meta-analysis findings. Leave-one-out analysis showed that excluding any single study did not change the statistical significance of the pooled effect, with ORs ranging from 1.91 to 2.43 (all *P* < 0.05). This stability indicates that the core conclusion linking elevated TyG-BMI to increased GDM risk is not driven by any individual study. Notably, exclusion of the study by Pinghua Li (23) led to a prominent reduction in heterogeneity from *I*^2^ = 97% to 60% and a strengthened pooled OR of 2.43 (95% CI: 1.84–3.21). This result identifies that study as the main source of the initial high heterogeneity, which may be related to its retrospective design, small sample size of 600 participants, and unique population features including a higher rate of advanced maternal age. These factors may have introduced selection bias and unstable effect estimates. The persistence of significant associations across all sensitivity analyses supports the reliability of TyG-BMI as an early predictive marker for GDM, even with some remaining heterogeneity in subgroups. Residual heterogeneity may be caused by unmeasured factors such as ethnic diversity, different GDM diagnostic criteria including International Association of Diabetes and Pregnancy Study Groups (IADPSG) one-step and two-step methods, and variable adjustment for confounders. These issues deserve further exploration in future research.

### 4.1 Strengths and Limitations

This meta-analysis exhibits several notable strengths: it systematically synthesized data from 6 high-quality cohort studies involving 52,877 participants, which enabled the investigation of the association between early-pregnancy TyG-BMI and GDM risk. The inclusion of both prospective and retrospective designs, as well as Chinese and Korean populations, enhances generalizability across diverse clinical settings and ethnic subgroups. Rigorous subgroup analyses and leave-one-out sensitivity analysis supported the stability and consistency of the results, confirming that the core conclusion was not overly dependent on individual studies. Notably, focusing on early-pregnancy TyG-BMI addresses a critical clinical gap, as current GDM screening is performed at 24–28 weeks. Its calculation relies on routine prenatal measurements (fasting triglycerides, glucose, and pre-pregnancy BMI), requiring no specialized testing and offering high translational potential in resource-limited settings.

Despite these strengths, several limitations should be acknowledged. First, significant heterogeneity (*I*^2^ = 97%) was observed across studies, which may be attributed to clinical and methodological differences including variations in GDM diagnostic criteria, gestational weeks at TyG-BMI measurement, inconsistent confounder adjustment, and diverse population characteristics. These clinical factors may represent the main sources of heterogeneity rather than statistical grouping alone. Second, the small number of included studies (*n* = 6) limits the statistical power of this meta-analysis; thus, all findings should be interpreted as exploratory. Publication bias assessments including funnel plots and statistical tests have limited reliability when fewer than 10 studies are included and must be interpreted with caution. Third, although a dose-response trend was observed in the synthesized data, the lack of individual participant data prevented the determination of a unified, clinically actionable cut-off value for TyG-BMI, which restricts the translational application of our findings. Fourth, the inclusion of only Asian populations limits the generalizability of the results to other ethnic groups. Finally, the three retrospective studies included carried inherent risks of selection bias, though sensitivity analysis indicated consistent results. Future research with large sample sizes, standardized protocols, diverse ethnicities, and individual participant data is warranted to validate these findings, explore clinical heterogeneity, and establish clinically applicable cut-off values for TyG-BMI in GDM prediction.

## 5 Conclusions

In summary, this exploratory meta-analysis of six cohort studies suggests that elevated early-pregnancy TyG-BMI is significantly associated with an increased risk of GDM. Although notable heterogeneity was observed, subgroup and sensitivity analyses support the reliability and consistency of this association. As a composite index incorporating triglyceride, glucose, and BMI, TyG-BMI holds considerable promise as a straightforward, cost-efficient early predictor of GDM risk in clinical settings. Future studies are needed to determine optimal and clinically applicable cut-off values of TyG-BMI for GDM prediction. Future large-scale prospective cohort investigations with standardized exposure measurement and unified diagnostic criteria are essential to further elucidate this association, identify potential effect modifiers, and ultimately advance early risk stratification and preventive strategies for GDM.
